# On Uterine Pains and Hemorrhage after Delivery

**Published:** 1855-11

**Authors:** W. Owen Brown

**Affiliations:** Caen, Corresponding Member of the Medico-Chirurgical Society of Bruges; Providence, R. I.


*On Uterine Pains and Hemorrhage after Delivery.* Translated from the French of Dr. Liegard, of Caen, Corresponding Member of the Medico-Chirurgical Society of Bruges, by W. Owen Brown, M. D., of Providence, R. I.*

There is a form of suffering to which newly-confined women are usually exposed denominated *uterine pains.* These pains are regarded by physiologists and accoucheurs as natural and necessary; and for their relief, as we have before said, a few insignificant remedies only, of which the experience of each day shows the inefficacy, are mentioned by authors. But if in most cases these pains are moderate, and if we may be allowed the expression, confined within physiological limits, it is not so with a great number of women, especially those who are of a feeble constitution and have already borne many children. It is unhappily only too common to see in these cases the pains prolonged for several days with great violence, and the patient left enfeebled and exhausted at the approach of the milk fever, which is thus rendered more troublesome and grave.

All accoucheurs have remarked that primiparæ suffer much less from these pains than others; it has even passed into a proverb with matrons that it is necessary to have them either *before* or *after* delivery; for they say women suffer much more before their first confinement than before the succeeding ones, and for this reason their sufferings are shorter after it—beautiful explanation, which explains nothing at all.

Accoucheurs and physiologists have assigned, as the principal cause of these pains, the obstruction offered by the contraction of the neck of the uterus to the escape of clots. But why are we to suppose this to offer more obstruction after the third or fourth labor than after the first? Observation and reason, on the contrary, demonstrate that the obstruction ought to be less considerable in proportion as the uterine neck has been often relaxed and distended by the escape of the fœtus. Some obstetricians have admitted a want of contraction in the uterine fibres; but they have deduced no consequences from these premises; they have not indicated any means by which they have been able to restore to these relaxed fibres their energy and contractility, so much have they been accustomed to regard these pains as natural and necessary. However this may be, this last explanation is perfectly conformable to sound physiology and experience.

Why then do primiparæ experience, after the expulsion of the placenta, so much less severe pain, and particularly of so

* The first part of this paper was published in the October number of this Journal from the same source.

much shorter duration? It is because the walls of the uterus have as yet been distended only by a single gestation, and possess much more energy than when, after many parturitions, they have lost their tonicity and contractility, they contract firmly upon themselves, and the uterus easily disgorges itself and expels from its cavity the blood *before it has time even to collect there and form coagula.* But in succeeding confinements, and particularly in women of a lymphatic temperament and feeble constitution, and especially in those confined with twins (which goes far to establish our theory that the uterine fibres possess but very feeble contractility) the blood accumulates in the womb, and there forms clots, which are expelled slowly and painfully, with violent and persistent uterine contractions. If, therefore, the surgeon had at his command means sufficiently powerful to restore to the body of the enfeebled uterus its energy and its contractility, it would certainly spare the woman those long pains which cause her so much bitterness at a moment which should be so grateful to her. But these means are precisely the same as I pointed out in the first part of this memoir, and which succeeded perfectly, and always, as we have seen, when it was wished to prevent a *perilous loss of blood after confinement*—I mean the ergot of rye, given *immediately before* the expulsion of the child, and *cold injections into the umbilical vein in order to effect the detachment of the placenta.*

The causes which produce hemorrhage after accouchement are also those which predispose to the persistence of *uterine pains;* and it would seem that *a priori* we should advise recourse to the same successful mode of treatment. I may state, however, that observation alone has conducted me to this valuable discovery, and that I made it in pursuing my observations upon the means of guarding against these hemorrhages. The first observation is, therefore, relative to a woman evidently threatened with grave hemorrhage after accouchement:

Case I.—April 11, 1847, at 5 P. M., I was called to a woman who had been having labor pains about three hours. She was at the term of her ninth pregnancy. She told me that all her labors had been very easy and fortunate, but that all, and *especially the last,* had been followed immediately by a considerable loss of blood, and by violent pains lasting for two or three days, with the expulsion of clots of blood, which had extremely fatigued her and left her feeble for more than a month after. The waters began to flow early in the evening. It was a head presentation with the occiput in advance; the neck of the womb was entirely dilated; the pains, though feeble, recurred every three minutes, and made the perineal tumor very prominent. Everything, therefore, announced that the labor would soon terminate. However, an hour after, things remained in the same state. I administered one gramme and a half of the ergot of rye (about 25 grains.) The pains became more energetic a quarter of an hour after, and at 7 o'clock the head was at the point of passing the vulva. I repeated the dose of ergot; and some minutes after, the child, strong and well-formed, was expelled. The uterus remained sensibly contracted upon the placenta, but no pains were at first experienced. It was not until half an hour had elapsed that some slight alternating contractions became manifest. Traction upon the cord was, however, made without result. I then injected some cold water into the umbilical vein, and almost immediately a cold sensation was experienced in the uterus, which contracted strongly, and two minutes after slight traction upon the cord brought away the placenta without resistance. The womb maintained a state of *continued* contraction for at least half an hour, and then moderate expulsive efforts, scarcely painful, began to be experienced at long intervals, denoting the escape of a little blood; no clots were expelled. She passed a comfortable night. The following morning the skin was moist; the sanguineous lochia were less abundant, and already a little serous. The mother nursed her child during the day, and eat two soups. The *uterine pains were insignificant.* The patient rose on the seventh day.

I was impressed in this case with the mild character of the uterine pains, and their short continuance; the woman herself was surprised. After having reflected, therefore, deliberately upon all the circumstances of the case, and after having recalled other similar examples in which the same means had been followed by the same results, I came to the resolution for the future to excite uterine contractions not only in order *to avoid dangerous hemorrhage after the escape of the placenta*, but also to *prevent, or greatly to diminish, uterine pains.* It has been remarked, as I said above, that a twin birth was followed by longer-continued and more severe pains, but no one has sought as far as we know to explain the cause. Dr. Windrif reported in the Medico-Chirurgical Journal for December, 1849, a very interesting case of superfœtation. It occurred to a lady who was confined on the same day of two children, one of which was at term, the other at 7 months, and he added, "though this was a first confinement, the mother had very strong pains, which continued for fifteen hours after delivery, with the expulsion of clots and abundant lochia." Thus this author confirms two things—1st, longer-continued and more severe uterine pains after twins; 2d, that the ordinary pains attending a first confinement are less persistent than those attending succeeding ones. He evidently recognized, by the astonishment which it caused him, the prolongation of these pains, but he did not attempt to account for the phenomenon.

CASE II.—At the end of November, 1847, Mrs. D—— was delivered naturally, after a labor of fifteen hours, of a strong, well-formed boy. The uterus afterwards remained very distinctly developed, and evidently contained a second fœtus, but the uterine contractions were suspended for twelve hours. Then after a renewed labor of three hours a second bag of waters became very prominent and was opened, and soon a fœtus was expelled. It bore all the signs of death dating several days prior to the birth. This lady informed us that eight days previous to her confinement she had met with a fall from her stairway, in which the right side of the abdomen had been violently struck. Thus during all this time the uterus was able to contain a healthy, well-formed child, and one for several days dead. It only concerns us to state that the uterine pains presented a persistence and a violence very extraordinary. They ceased only on the third day, despite the application of emollients and narcotics, employed perseveringly and in large doses. This was the third labor with this woman. The pains in other cases, where there has been but one child, had seldom persisted more than twelve or fifteen hours.

Case III.—April 16, 1847, at 9 P. M., I was called to a woman who had been having labor pains for several hours. For the last half hour they had been very severe, and the labor was near terminating. In short, hardly twenty minutes after my arrival, I received a very strong child, which immediately began to cry sharply. This was the seventh time I had assisted this woman upon similar occasions, and I knew that the placenta always detached itself slowly and with difficulty, because of the feeble uterine contractions, and that its extraction was followed by a considerable loss of blood, and by very severe and prolonged pains. In the two last accouchements, particularly, the pains had continued for three days and three nights, during which time she had been unable to obtain either quiet or sleep. Twenty-five minutes after the escape of the child some slight pains began to be felt. I made feeble traction upon the cord; the placenta was adherent, and I at once desisted, and immediately threw a cold injection into the umbilical vein. A sensation of coldness manifested itself in the uterus, and almost immediately this organ began to contract. The contractions gradually increased, and the placenta was expelled by the aid of slight traction. Afterwards there escaped some spoonsful of red, fluid blood. The womb remained contracted, without pain, and under the influence of this prolonged but not painful contraction, its volume gradually diminished and sleep was refreshing. Only a few very distant pains were perceived towards morning, and they disappeared entirely in the course of the day. This woman nursed her child; she was up on the fifth day, 21st April, and her health and strength were immediately re-established.

In this case we have at once a double success—both the absence of hemorrhage and of uterine pains, the occurrence of which the experience of her former labors would almost certainly indicate.

Case IV.—Mrs. E. D., aged 35, of a sanguine, lymphatic temperament, and a good constitution, had borne four children. I attended her in her two last confinements, and in both instances the uterine pains following were violent and prolonged for two or three days, so that the patient expressed her sufferings as being greater subsequent to, than preceding, her confinement. At the last, particularly, the pains continued through two days and nights, so as to entirely deprive her of sleep. In the month of January, 1848, some years after her fourth confinement, she found herself at the term of her fifth pregnancy. In the night of the 20th or 21st, after one feeble uterine pain, the membranes ruptured, and the waters flowed abundantly; this was succeeded by a long repose. At 6 o'clock the contractions became very energetic and regular, and at 8 o'clock a strong girl was born naturally, and without the least accident. Ten minutes after a slight contraction manifested itself; the placenta, however, remained firmly adherent. I then injected 400 grammes (about 12 fluid ounces) of cold water into the umbilical vein. The uterus contracted at once strongly, and the placenta was removed by slight traction upon the cord. During the two hours next following, the pains succeeded each other at short intervals, accompanied by abundant sanguineous lochia, which disgorged the uterus and diminished its volume considerably. These pains became gradually slighter until afternoon, and during the night she was almost completely exempt from them. On the morning of the 22d, they returned suddenly. The patient having been placed upon a night-vessel to urinate, a large clot was expelled, and from that time the pains, were scarcely felt, and disappeared entirely thirty hours after confinement.

Case V.—Mrs. Germain, aged 26 years, of a nervous, sanguine temperament, was confined with her third child June 14th, 1848, at noon, after a labor of two hours and a half, *with good pains* (at her second accouchment, 16 months before, the uterine after-pains had persisted for three days.) At twenty minutes past 12, no uterine contraction announced the approaching expulsion of the placenta, which remained completely adherent, and I made an injection of 120 grammes of cold water into the umbilical vein. A slight sensation of coldness was produced in the uterus. Four minutes after, things remaining in the same state, another injection was made. The sensation of coldness was greater, and the womb contracted energetically. Slight traction was made upon the cord, which brought away the placenta, the lobules of which were a little cold, evidently infiltrated by the liquid injected. The womb remained contracted during the entire afternoon, but there were no uterine pains. The crying of the child, during the night, kept the mother almost constantly awake; but the following morning she did not complain to me of any after-pains; and it was not until I had particularly questioned her regarding it, that she told me it was true she had felt some colic pains, but very slight. She experienced a few more pains during the 15th; but the following night, after having nursed her child, it was removed to another apartment, and she slept soundly and felt no more of the pains. On the 16th, and the following days, the lochia flowed in the usual manner. On the 17th she sat up four hours. She was able to walk out on the eighth day.

In this case, as in all others where I have not anticipated hemorrhage, I have employed injections only. In the following case I arrived too late to employ the ergot before the delivery of the child; but it will be seen that by the injections alone, I succeeded equally well. I believe, therefore, it is possible to attain our purpose with this means alone, but yet prudence ought to lead us to adopt the two modes of treatment in grave circumstances.

CASE VI.—I was called at 2, P. M., Oct. 7, 1848, to go immediately to visit a woman who was the subject of the first observation related in this second part of my paper. The case was a very urgent one. I found, on my arrival, that the child had been expelled some minutes (it was her tenth confinement.) I hastened to ligate and divide the cord; then I waited about ten minutes without any uterine contractions becoming apparent. This woman spoke to me of her fears of hemorrhage and after-pains, to which she had been subject. I encouraged her by reminding her of the success of her last accouchement, and informed her that I had since been successful in many other cases. I threw three successive injections into the umbilical vein, each of 145 grammes of water at the common temperature (it was a warm day;) after the second, there was experienced a slight sensation of coldness; after the third, uterine contraction was energetic. Feeble traction sufficed to bring away the placenta; it was a little cool in all its parts, and very much distended by the water injected. I remained about an hour after this with the woman. There was no hemorrhage: the womb remained firm, without any painful contractions. * * The following morning she assured me she had experienced *no colic*. She had no fever, had already given the breast to the child several times, and the uterus was much less voluminous than on the evening before. The lochia were slightly sanguineous and flowed well. This woman rose on the seventh day, and now (Dec., 1848) the mother and child are in good health.

Case VII.—An English lady was taken with labor pains on the morning of the 25th October, 1848. It was her second confinement, the first having occurred seven years before. The child was born without instrumental aid at 3 o'clock, P. M., after two hours of very hard pains. As this lady had met with a very alarming loss of blood after her first confinement, I administered two grammes of the ergot of rye, near the termination of the labor, and, in order to detach the placenta, I injected 150 grammes (about 5 fluid ounces) of cold water into the umbilical vein. The cord was very short, and this single injection excited immediately a sensation of cold in the uterus, and produced the expulsion of the placenta, which was not succeeded by hemorrhage or after-pains. Lochia natural and free. This lady was up on the seventh day.

I could relate many similar cases, but it is useless to do so when reason and sound physiology so clearly demonstrate the principles which it is intended to illustrate. In all these cases, after employing the means I have indicated, the labor progressed precisely as does that of a first accouchment. The uterine walls opposed each other firmly, and prevented the accumulation of blood, and the formation of coagula. The after-pains, sometimes absent, were in other cases quite severe, but soon diminished, and disappeared entirely. The flowing of the sanguineous lochia was unobstructed and quite easy, less in quantity and continued a shorter period of time than when the injection was not used. In fact, in place of being prolonged to 30 or 40 hours, the lochia were seen to diminish 10 or 12 hours after the accouchement; the vermillion color gradually grew less, and after the second day there was not more than a reddish serosity, which soon constituted what is called the *serous lochia.* This last secretion, in its turn, was natural and without pain, and was soon succeeded by the white discharge, which is more prolonged and more abundant, if the woman does not nurse her child, and if she has not the care of attending it. In this case, a less nourishing regimen, and one less less exciting to the secreting and depurating organs, and to the perspiratory system, is generally indicated. The lochia are affected, too, by age, temperament, season, climate, &c.

Physicians have formerly made long and ridiculous calculations respecting the quantity of blood a woman ought to lose after parturition, in order that the system may be *sufficiently purged.* The blood that a woman loses after the expulsion of the placenta, uselessly enfeebles her, if it flows in the absence of uterine contractions; it takes from the vital forces, of which she will have indispensable need, in performing the important function of nursing her child. There is no further flow of blood needful than that which accompanies the uterine contractions, and which serves to disgorge the walls of this organ, and to restore them to their normal or physiological limits. It is conceived, then, that some hundreds of grammes are amply sufficient; now by the means which we counsel, this double result is obtained promptly, and almost without pain.

As to the mode of making these injections, since it is so readily understood, I have not entered into a very detailed description. It will be well to have a syringe containing at least 150 grammes (4 or 5 fluid ounces) having a long, fine nozzel, or canula. Before introducing it into the vein make a clean section of the cord, for the purpose of distinctly seeing the vessels. The cord should not be, at the most, more than 12 or 15 inches long. In my first trials I used water acidulated with vinegar, especially when I anticipated hemorrhage. For some years past I have employed only simple cold water, and its action has appeared to me sufficiently powerful.

Whenever a dissection has been made of a placenta, detached by injection into the umbilical vein, it has been remarked that wherever the ramifications of the vessels have been followed, the liquid injected has been met with. The two faces of the placenta present a very different aspect. The internal or fœtal face is traversed throughout its extent by the transparent divisions of the vein distended by the cold water; the uterine surface, on the contrary, is red and injected by the blood contained in it. The liquid of the injection does not penetrate to the external surface; hence the temperature of this surface is found more elevated than the other, and the sensation of cold experienced by the woman is not so great as, *a priori*, would have been supposed. It is important that the sensation of cold in the uterus should be felt, since it announces, and determines, the uterine contractions, indispensable to the success of the operation. It is evident that if the water be very cold, a much less quantity will need to be injected; at the common temperature, in winter, 150 grammes will often suffice, while in summer two or three times that quantity may be required—*Boston Med. & Surg. Journal.*

